# Early-Expressed Chemokines Predict Kidney Immunopathology in Experimental Disseminated *Candida albicans* Infections

**DOI:** 10.1371/journal.pone.0006420

**Published:** 2009-07-29

**Authors:** Donna M. MacCallum, Luis Castillo, Alistair J. P. Brown, Neil A. R. Gow, Frank C. Odds

**Affiliations:** Aberdeen Fungal Group, Institute of Medical Sciences, Aberdeen, United Kingdom; Columbia University, United States of America

## Abstract

**Background:**

The mouse intravenous challenge model of *Candida albicans* infection is widely used to determine aspects of host-fungus interaction. We investigated the production of cytokines in the kidneys and spleen of animals up to 48 h after challenge with virulent and attenuated isolates and related these responses to semi-quantitative estimations of histopathological changes in the kidney.

**Methodology/Principal Findings:**

Progression of *Candida albicans* infection of the kidney in response to highly virulent fungal strains was characterized by higher levels of host cellular infiltrate, higher lesion densities and greater quantities of fungal elements at 24 and 48 h, and by higher kidney concentrations of IL-1β, MCP-1, KC, IL-6, G-CSF, TNF, MIP-2 and MIP-1β, among the immune effectors measured. Levels of the chemokine KC as early as 12 h after challenge correlated significantly with all later measurements of lesion severity. Early renal IL-6 and MIP-1β concentrations also correlated with subsequent damage levels, but less significantly than for KC. All chemokines tested appeared in kidney homogenates, while most of the cytokines were undetectable in kidney and spleen homogenates. GM-CSF and IL-10 showed inverse correlations with measures of lesion severity, suggesting these alone may have exerted a defensive role. Spleen levels of KC at all times showed significant associations with kidney lesion measurements.

**Conclusions/Significance:**

Elevated chemokine levels, including KC, represent the earliest responses to *C. albicans* infection in the mouse kidney. Fungal strains of low mouse virulence stimulate a lower innate response and less host infiltrate than more virulent strains. These findings are consistent with immunopathological damage to kidneys in the mouse *C. albicans* infection model and with growing evidence implicating some TLR pathways as the main point of interaction between fungal surface polysaccharides and leukocytes.

## Introduction

Disseminated *Candida albicans* infections are often difficult to diagnose and are associated with a high mortality [1], [2]. Considerable research effort has gone into understanding fungal virulence factors [3], [4], [5] and host immunity [6], [7], [8] in experimental *C. albicans* infections in mice. For very many years, the mouse model for disseminated *Candida* infection has involved intravenous (IV) injection of a yeast inoculum, with time to mortality and viable fungal burdens in tissues the main markers of differences in virulence of fungal strains and mutants, or of differences in mouse susceptibility to fungal challenge [9], [10], [11].

The kidney is the principal target organ for infection in the mouse IV challenge model and, while sepsis has been evidenced as the major cause of death in the mouse model [9], the extent of kidney damage in animals showing severe symptoms is considerable and is likely to contribute to the overall pathology of the disease [9], [11]. It has been recognized for many years that the disease processes in *C. albicans*-infected kidneys result in heavy host leukocyte infiltrates and micro-abscess formation [12]. This process suggests a contribution of host immune responses to tissue damage in the kidney, a contribution long recognized [13] and well accepted [14] for many bacterial infections. Hence we set out to explain the pathological basis for kidney damage following *C. albicans* infection.

The kidney is not the only organ affected in the mouse *C. albicans* challenge model. Invasion of the brain by *C. albicans* occurs in animals receiving high challenge doses [11]. In the spleen, lungs and liver, viable fungi are gradually cleared even while infection damage progresses in the kidneys [10], [11]. Detailed studies of pathological events in the mouse model indicate that changes associated with disease become measurable within 3 days of challenge with *C. albicans*, including body weight, systolic blood pressure, blood glucose, urea, chloride and creatinine levels [9].

The mouse IV challenge model is a highly reproducible, long-standing, and widely used test for investigations into host-fungus interactions and efficacy of antifungal agents. However, evaluation of virulence effects solely in terms of kidney burdens and survival times seems a rather crude and unsophisticated approach against which to examine host immune responses when compared to current technologies which permit determination of levels of individual cytokines, enumeration of leukocytes of different receptor types and generation of RNA expression profile data for host and fungal cells.

Consistent experimental evidence indicates that early innate immune responses, rather than adaptive responses, are essential for protection of mice against IV *C. albicans* challenge [7], [8], [15] and progress has been made *ex vivo* and *in vivo* towards identification of specific interactions between leukocyte receptors involved in innate responses and different surface polysaccharides in *C. albicans* — the so-called ‘pathogen-associated molecular patterns’ (PAMPs) [16]. Spellberg and colleagues concluded that failure of the kidney to halt progression of *C. albicans* infection correlates with cytokines produced locally in the infected organ, rather than with systemic immune responses, as represented by cytokine production in splenic cells [10]. The long-standing view has been that overall protective vs. non-protective immunity to *C. albicans* challenge depends on a response dominated by Th1 rather than Th2 cells (and this response is at least partly a function of mouse genes *Carg1* and *Carg2* [7]). However, recent work by Romani's group suggests that IL-17-producing cells, induced subsequent to the immediate innate responses to *C. albicans*, promote damaging inflammation and impair the anti-*Candida* effects of neutrophils at sites of gastric infection (reviewed in [8]).

*C. albicans* yeast cells that have been transported from the bloodstream into visceral organs in experimentally infected mice can be thought of as encountering a changing microenvironment with progression of time. At first the fungi engage with a succession of innate-immunity leukocytes then later lymphocytes, all involved in a complex chemical cross-talk based at the earliest stages on production of a mixture of chemokines and cytokines by host leukocytes, and of surface polysaccharides and secreted proteins by the fungus. At any temporal stage, the balance between elimination of the fungal cells and local damage at the site of infection will depend on the responses both of the leukocytes and the fungus to this array of effector molecules and cells. When an immune response is characterized as predominantly Th1 or Th2, this reflects the overall outcome of the fungus-host interaction, but does not indicate the component events that led to that outcome.

We hypothesized that a more detailed picture of the host-fungus interactions that occur in a kidney infected with *C. albicans* could be obtained by measurement of a greater number of chemokines and cytokines than has previously been attempted, and by semi-quantitative histopathological analysis of the lesions. By infecting mice with a set of *C. albicans* strains chosen to represent examples known to be of high and low virulence in the mouse model we aimed to differentiate host responses and lesion parameters that correlate with survival and non-survival of the experimental infection. Because the available evidence suggests that the early stages of host-*C. albicans* interactions determine gross clinical outcomes, we confined our monitoring of events to the first 48 h after IV challenge.

## Results

### Quantitation of lesions: associations with strain virulence

**Figure 1:**
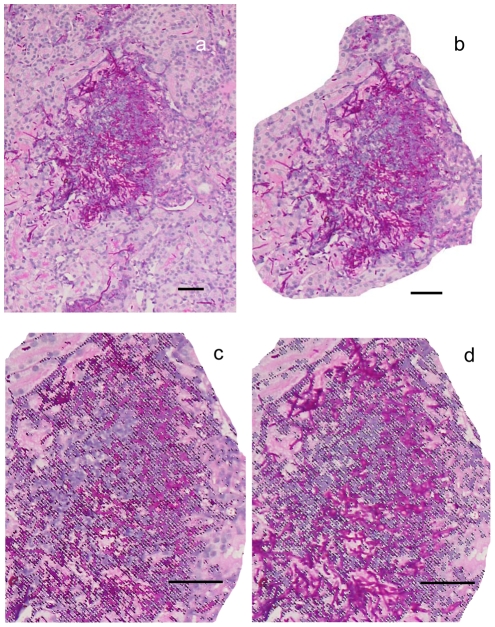
PAS-hematoxylin-stained kidney section showing *C. albicans* elements and host infiltrates. a. Photograph of kidney section. b. Lesion isolated, copied and pasted in Adobe Photoshop. c. Higher magnification of (b) showing magic wand selection of PAS-positive pixels. d. As c but with host infiltrate pixels selected. Bar = 50 µm.

**Figure 2:**
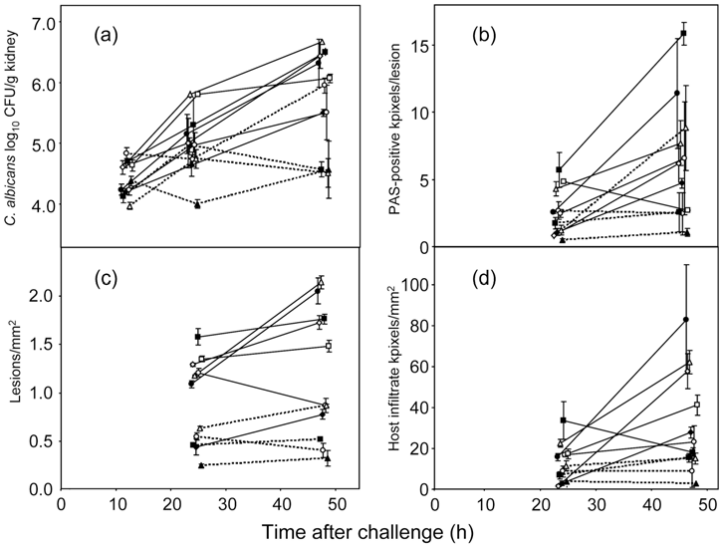
Development of kidney lesion parameters over 48 h from intravenous challenge of mice with 11 *C. albicans* strains. Data show means±SEMs for data from 3 mice per time point per strain. X-axis values have been jittered to facilitate visualization of the data. (a) Viable fungal burdens in kidneys. (b) *C. albicans* per lesion as PAS-positive pixels/lesion. (c) Lesion density (lesions/kidney section). (d) Host infiltrate pixels/mm^2^. Strains are depicted as follows. J990102, open circles; J951361, filled squares; AM2003-020, open triangles; IHEM16614, open diamonds; SC5314, closed circles; s20175.016, open squares; AM2003/0074, filled diamonds; AM2003/0069, open circles with dotted lines; HUN96, filled squares with dotted lines; *Capmr1*, open triangles with dotted lines; *Capmr1*+*PMR1*, filled triangles with dotted lines.

Our first objective was to evaluate how well the three new semi-quantitative histopathology measurements, viz. lesion density, *C. albicans* pixels per lesion and infiltrate pixels per mm^2^ kidney (Fig. 1), compared with viable kidney burdens, the conventional measurements of infection progression in the mouse model. Fig. 2 shows the data for the four lesion parameters at 24 and 48 h, and, additionally, at 12 h for viable kidney burdens. Error bars were often substantial because only 3 mice were analyzed per time point for each strain. The data showed an increase in each measurement from 24 h to 48 h for the majority of infecting strains. Two of the least virulent strains, HUN96 and *pmr1*Δ (Table 1) showed a decrease in viable kidney burdens between 24 and 48 h (Fig. 2a), while the highest burdens at 48 h were for AM2003-020, J951361, IHEM16614 and SC5314 (Fig. 2a), which were 4 of the 5 most virulent strains in a previous study (Table 1). The highest densities of PAS-positive pixels, representing fungal material in the kidney lesions (Fig. 2b), lesion densities (lesions/mm^2^ section; Fig. 2c) and host infiltrate pixel densities were again all recorded for the most virulent strains in the panel (Table 1). Thus overall strain virulence was related not only to viable kidney burdens, but also to the density of kidney lesions, the amount of host leukocyte infiltrate and the amount of PAS-positive material as measured histopathologically. The virulence rank scores for the 9 strains studied previously (Table 1) showed strong statistical correlations with the 48 h means of these four parameters (Fig. 2): mean viable burdens, *r* = −0.67 (*p* = 0.025), mean kidney lesions/mm^2^, *r* = −0.74 (*p* = 0.011), mean PAS-positive pixels in PAS-positive lesions, *r* = −0.64 (*p* = 0.031), and mean host infiltrate pixels/mm^2^, *r* = −0.52 (*p* = 0.078).

**Table 1:** Details of the 11 *C. albicans* strains used in this study.

Isolate no.	Virulence	MLST data		Country	Source	ABC	MTL	Survival time (days)	Kidney	Weight change
	Virulence rank^a^	Clade	DST	of isolation	of isolate	type	type	Mean±SD^b^	Log CFU/g (Mean±SD)^b^	day 0–3 (Mean±SD)^b^
J990102	1	3	45	Belgium	vagina	B	*a/α*	6.5±0.8	7.1±0.3	−10.3±4.3
J951361	5	1	44	UK	blood	A	*a/α*	5.3±1.6	6.1±1.6	−15.1±4.6
AM2003-020	7	4	124	UK	oropharynx	C	*a/α*	13.2±8.9	7.1±0.8	−9.9±4.4
IHEM16614	8	2	206	Rwanda	oropharynx	A	*a/α*	7.3±1.4	6.4±0.6	−9.9±3.3
SC5314	9	1	52	USA	blood	A	*a/α*	6.8±1.6	6.0±1.0	−15.7±2.2
s20175.016	20	4	124	Israel	blood	C	*a/α*	26.2±3.0	6.9±1.2	−7.0±5.9
AM2003/0074	34	3	198	UK	blood	B	*a/α*	27.3±1.6	6.0±1.2	1.1±5.0
AM2003/0069	35	2	194	UK	vagina	A	*a/α*	24.5±4.2	4.9±1.7	0.7±3.2
HUN96	42	1	116	UK	blood	A	*a*/*α*	28.0±0.0	4.0±2.2	1.7±3.6
NGY355 (*pmr1*Δ)					SC5314			28.0±0.0	2.9±2.0	−1.5±2.0
NGY356 (*pmr1/PMR1*)					SC5314			16.2±7.2	5.1±1.8	−9.2±1.0

a among 43 isolates from prior study [37]. The 43 isolates were separately ranked for mouse virulence by mean survival time, terminal viable kidney burdens and weight change from day 0 to day 3, and the ranks combined to give the overal placing among the 43 isolates as shown in this table. The first five isolates listed therefore represent highly virulent strains, and the next four represent isolates of low virulence.

b historical data [37].

### Lesion parameters correlate with strain virulence at different times after challenge

**Figure 3:**
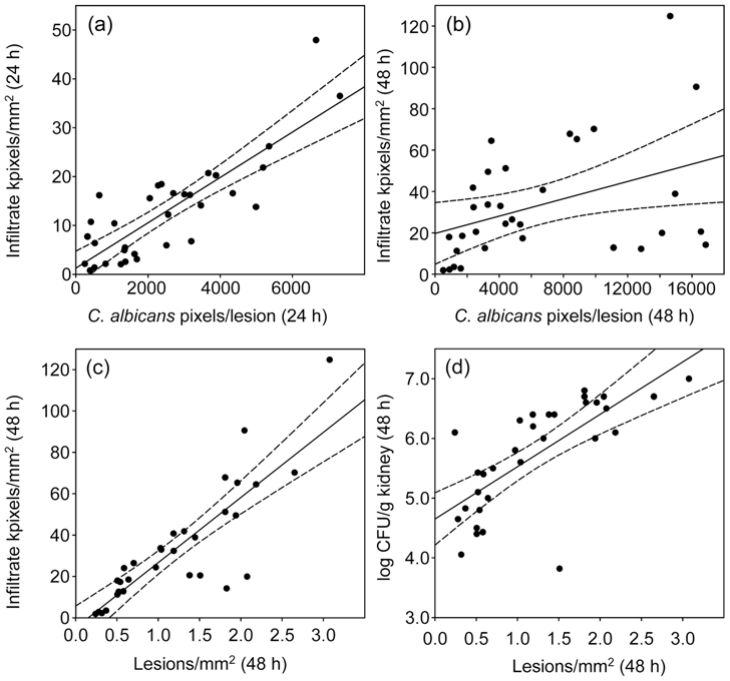
Scatterplots of lesion parameters. Each point represents the result for a single animal, with 3 animals per *C. albicans* isolate. In each figure the solid line is the regression line and the dashed curves indicate the 95% confidence region. (a) Lesion density (48 h) vs. *C. albicans* kidney burdens (48 h). (b) Lesion density (48 h) vs. infiltrate density(48 h). (c) *C. albicans* pixel density (24 h) vs. infiltrate pixel density (24 h). (d) *C. albicans* pixel density (48 h) vs. infiltrate pixel density (48 h).

Fig. 3 shows examples of strong and weak cross-associations between the data obtained by measurements of lesion parameters for kidneys infected with 11 isolates of *C. albicans*. The data available from measurements at 24 and 48 h generated 36 scatterplots of the types illustrated in Fig. 3, by inclusion of 12 h viable burden data. These are summarized in Table 2 as cross-tabulations of correlations between the various measurements of kidney lesion histopathology and fungal burdens for animals infected with the 11 *C. albicans* strains. The correlations between the lesion parameters often reached statistical significance (Table 2). Overall, the results in Table 2 show that infection with the most virulent strains (Table 1) generated more lesions, more host infiltrate and greater quantities of fungal material, viable or not, than infection with the least virulent strains.

**Table 2:** Correlation coefficients (Pearson *r*) cross-tabulated for means of lesion data for 11 *C. albicans* strains.

	Time	Fungal kidney burdens			Lesion density		Infiltrate density		*C. albicans* pixels/lesion	
	(h)	12 h	24 h	48 h	24 h	48 h	24 h	48 h	24 h	48 h
Viable fungal burdens	12									
	24	0.41								
	48	**0.63**	**0.57**							
Lesion density	24	**0.55**	**0.59**	0.76*						
	48	0.40	**0.54**	0.84*	0.81*					
Infiltrate density	24	0.43	0.49	0.51	0.71*	**0.57**				
	48	0.12	0.36	0.73*	**0.53**	0.85*	0.16			
*C. albicans* pixels/lesion	24	**0.60**	0.73*	**0.62**	0.72*	**0.56**	0.90*	0.19		
	48	0.20	0.02	0.51	**0.63**	**0.67**	0.73*	0.35	0.48	

For each time point *N* = 33 samples (3 per infecting strain).

Numbers in boldface type indicate *p*≤0.05; an asterisk indicates *p*≤0.01. Data shown are all correlation coefficients, so the original units of measurement are not stated in the column headings. Data for fungal kidney burdens were log_10_ CFU/g kidney, and for lesion densities as lesions/mm^2^ and for infiltrate densities as pixels/mm^2^.

### Chemokine levels are the first to rise in tissues of mice infected with *C. albicans*

**Figure 4:**
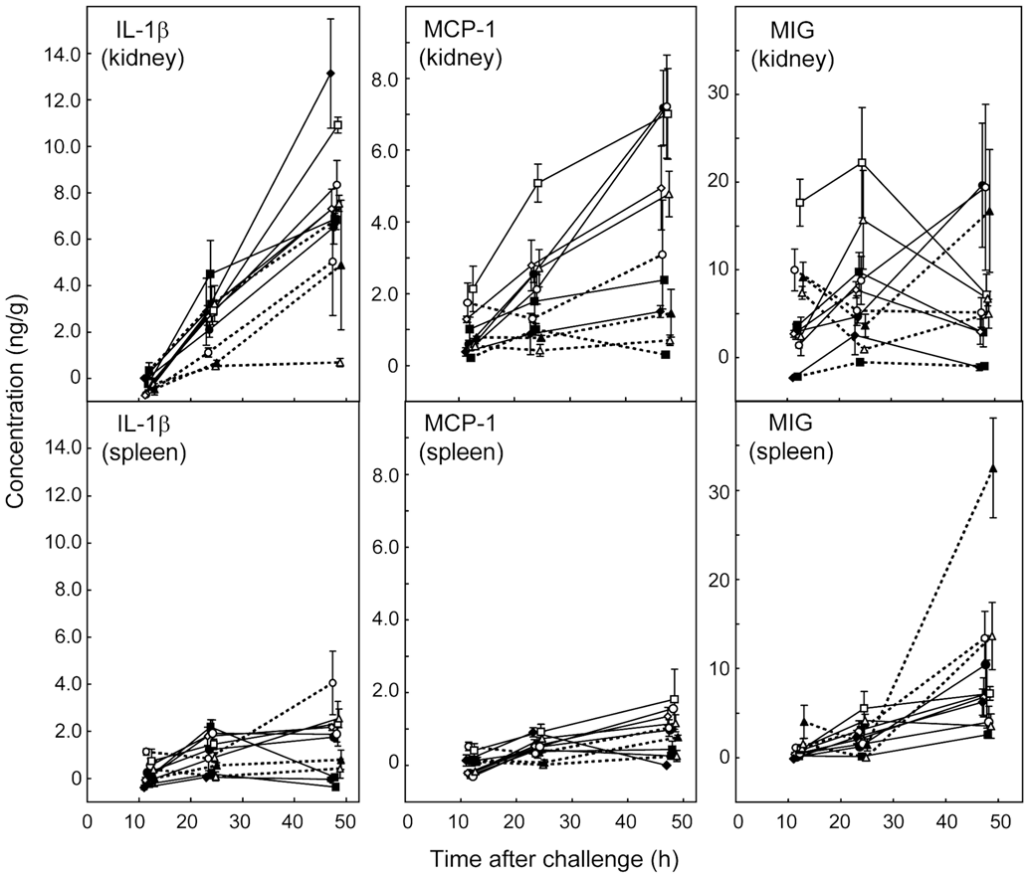
Time course of concentrations (ng/g) of the three most strongly concentrated cytokines or chemokines in supernatants from kidney homogenates after infection of mice with 11 *C. albicans* strains, together with corresponding data for spleen homogenates. The mean±SEM of 3 observations are shown for each time point measured (12 h, 24 h and 48 h). X-axis jitter has been used to aid visualization of the data points. For each immune effector, the scale for the kidney and spleen data is the same, to allow comparison of levels between the two tissues assayed. In each graph, the 11 strains are distinguished as follows: J990102, open circles, solid lines; J951361, filled squares, solid lines; AM2003-020, open triangles, solid lines; IHEM16614, open diamonds, solid lines; SC5314, filled circles, solid lines; s20175.016, open squares, solid lines; AM2003/0074, filled diamonds, solid lines; AM2003/0069, open circles, dotted lines; HUN96, filled squares, dotted lines; *Capmr1*, open triangles, dotted lines; *Capmr1 + PMR1*, solid triangles, dotted lines.

**Figure 5 pone-0006420-g005:**
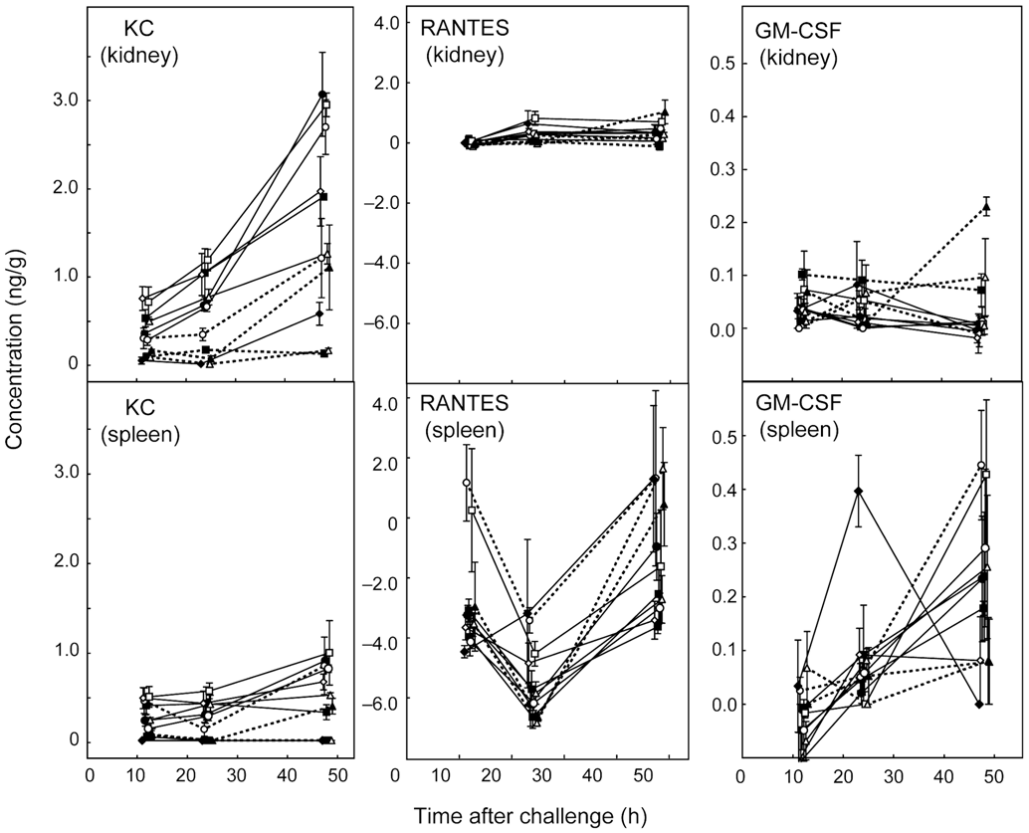
Time course of concentrations (ng/g) of KC, RANTES and GM-CSF in kidney and spleen samples from mice infected with 11 different *C. albicans* strains. Details as in Fig. 4.

Figs. 4 and 5 show the concentrations of the most abundant chemokines and cytokines in organ samples at 12, 24 and 48 h. Chemokines MCP-1 (Fig. 4), MIG (Fig. 4), and KC (Fig. 5) were abundant in samples from infected kidneys by 12 h after challenge. MIG was the most abundant chemokine at all time intervals in kidney samples (Fig. 4). However, while levels of KC and MCP-1 tended to rise in kidneys of mice infected with all 11 strains over the 48 h period of measurement, MIG levels rose between 24 h and 48 h only for animals infected with J990102, SC5314 and *pmr1/PMR1*. For the remaining isolates MIG concentrations were similar at 24 and 48 h or fell from a peak at 24 h (Fig. 4). Detectable levels of GM-CSF were measured at 12 h for some kidney samples (Fig. 5), but they remained low (below 100 pg/g on average) throughout the time course of the experiment with the exception of the 48 h kidneys from mice infected with *pmr1/PMR1*.

### Chemokine and cytokine concentrations tended to be higher in kidneys of mice infected with virulent strains

**Figure 6:**
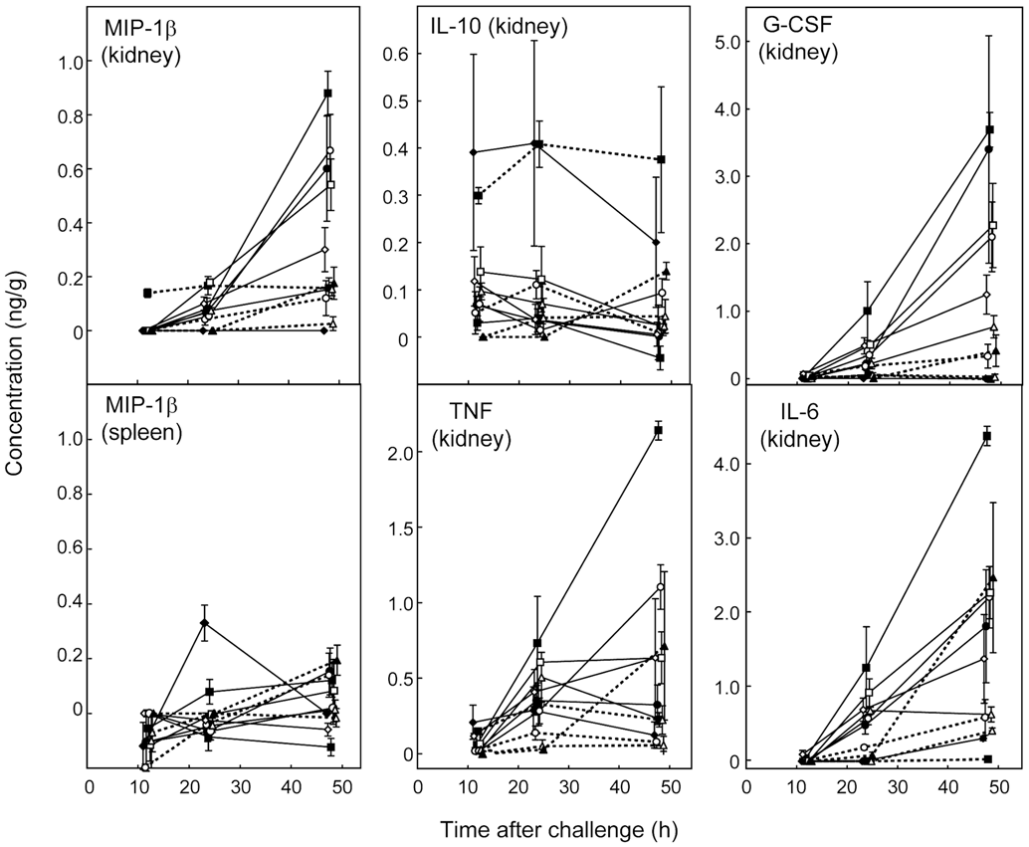
Time course of concentrations (ng/g) of the chemokine MIP-1β in kidney and spleen samples, and of four cytokines measured in kidney samples. Among the latter only IL-10 was measurable in spleen samples (see text). Details as in Fig. 4.

By 24 h after *C. albicans* challenge, kidney levels of IL-1β (Fig. 4), G-CSF, IL-6, TNF, and MIP-1β (all Fig. 6) had risen substantially. For these effectors, as well as chemokines KC (Fig. 5), MCP-1 (Fig. 4), and MIP-2 (where measured; see supplementary table S1), levels in infected kidneys by 48 h were generally highest for mice infected with the most virulent strains (Table 1). Among the kidney samples, data for IL-10 alone (Fig. 6) showed no consistent trend. Mean levels of IL-10 for kidneys from mice infected with *pmr1*Δ and AM2003/0074 were higher than for mice infected with the other 9 strains throughout the time course. At 24 h kidney levels of RANTES (Fig. 5) were also higher in mice infected with the more virulent strains, but this pattern was lost by 48 h. Among the 20 cytokines and chemokines measured in kidney samples 12, 24 and 48 h after challenge, IFN-γ, IL-2, IL-3, IL-4, IL-5, IL-9, IL-12p70, IL-13 and IL-17 levels were undetectable in more than 90% of kidney samples at any time. To confirm this unexpected negative finding we retested IFN-γ and IL-12p70 by immunoassay as many samples as residual homogenate volumes allowed, together with two homogenates spiked with IFN-γ and IL-12p70. The results estimated the spiked cytokine levels to within 20% of the spiked concentrations, but confirmed there were no detectable levels of the cytokines listed above in the native samples. In spleen samples, the same cytokines plus G-CSF, IL-6 and TNF were undetectable in more than 90% of samples. Hence data for these immune effectors were not analyzed further.

### Cytokine and chemokine levels in spleen differed markedly from those in kidney samples

In spleen samples from mice infected with 11 *C. albicans* strains, chemokine KC and cytokine IL-1β were present at easily detectable levels 12 h after challenge (Figs. 4,5), while other chemokines and cytokines reached more substantial concentrations by 24 and 48 h (Figs. 4–6). G-CSF, IL-6 and TNF, which were found in 24 h and 48 h kidney samples, were undetectable at all times in spleens. RANTES concentrations in spleen (Fig. 5) followed a time course different from all other cytokines and chemokines measured. Mean control levels of this chemokine in spleen from saline-injected mice were 4300 pg/g at 12 h, 7500 pg/g at 24 h and 6400 at 48 h. These values exceeded the concentrations measured for spleen samples from *C. albicans*-infected animals at all three times, indicating downregulation of this chemokine in response to infection. By 48 h, spleen RANTES concentrations from most infected animals were still lower than controls (Fig. 5) but showed recovery from their 24 h low values. The highest RANTES spleen levels at 48 h were measured for mice infected with the least virulent isolates.

By 48 h, GM-CSF levels in spleens were greater than those in kidney samples (Fig. 5). However, apart from GM-CSF and RANTES, the spleen chemokine and cytokine responses to *C. albicans* infection were of a lower magnitude than responses in the kidney (Figs. 4–6). MIG levels in spleen at 48 h were highest for mice infected with the lowest virulence strains, while MCP-1 levels in spleens at 48 h were highest for mice infected with the most virulent strains. Spleen levels of IL-10 (details not shown), like those of IL-10 in the kidney (Fig. 6) were generally low at all times, with means never exceeding 300 pg/g, and similar to the kidney samples, showed no clear trends over the 48 h period of measurement.

### Statistical associations confirm KC as an early predictor of kidney damage

**Figure 7:**
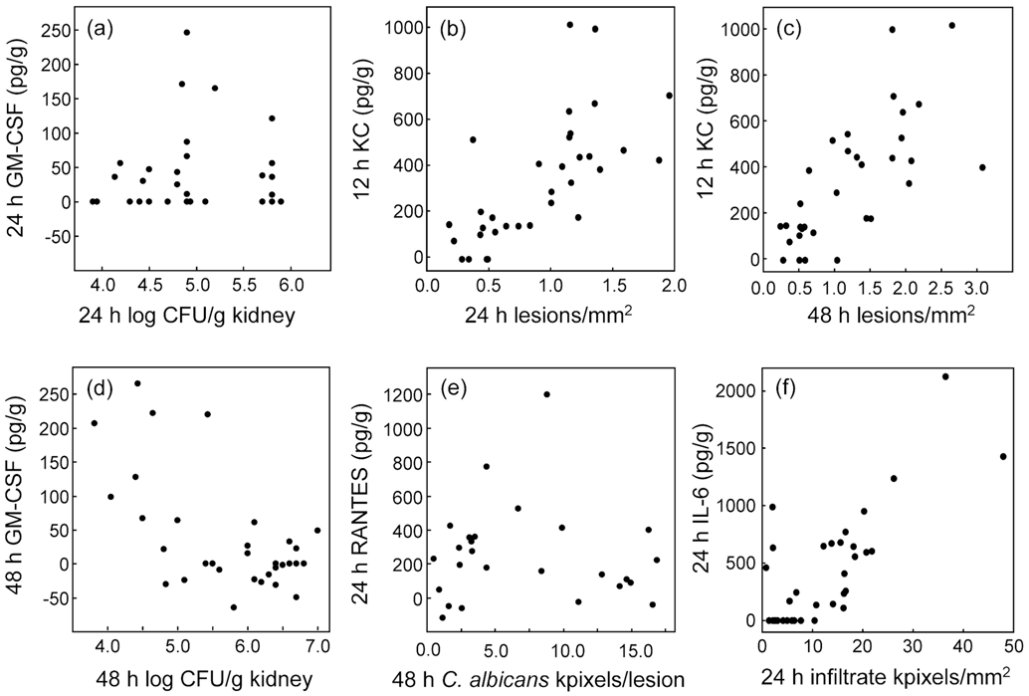
Scatterplots of cytokine or chemokine concentrations in kidney samples vs. lesion parameters for animals infected with 11 *C. albicans* strains: 3 points per strain. (a) GM-CSF at 24 h vs viable fungal burdens at the same time; *r* = 0.08, *p* = 0.33. (b) KC at 12 h vs lesion density at 24 h; *r* = 0.84, *p*<0.001. (c) KC at 12 h vs lesion density at 48 h; *r* = 0.67, *p*<0.001. (d) GM-CSF at 48 h vs viable fungal burdens at the same time; *r* = −0.61, *p*<0.001. (e) RANTES at 24 h vs mean *C. albicans* pixels per PAS-positive lesion; *r* = −0.05, *p* = 0.41. (f) 24 h IL-6 vs mean host infiltrate pixels per mm^2^ section; *r* = 0.77, *p*<0.001.

The tendency for higher levels of some cytokines and chemokines to develop in the kidneys of mice infected with the most virulent *C. albicans* strains (Figs. 4–6) was reflected in a statistical analysis of associations between measurements of immune effectors and lesion parameters. Fig. 7 shows examples of strongly and weakly associated lesion parameters and cytokine or chemokine levels.

The associations between immune mediators and lesion parameters were explored in detail by statistical analysis for significant correlations between cytokine or chemokine levels at each of 3 sample times vs kidney lesion parameters at 24 and 48 h (Table 3). Associations are shown both as Pearson's correlation coefficients (*r*) and as *F*-ratios from univariate ANOVA tests. Table 4 reduces Table 3 to a simpler list of associations, in which cytokine or chemokine measurements that showed 3 or fewer statistically significant associations with lesion data at all measurement times have been omitted. The values of *r* and *F* have been replaced with a score from 0 to 4 that indicates the statistical strength of each association. In this system, 0 indicates no statistically significant association, and the score is increased by 1 for each *r* or *F* value significant at p<0.01, and by 2 for each *r* or *F* value significant at *p*<0.001.

**Table 3:** Statistical associations between cytokine and chemokine measurements in kidney and spleen samples, measured at 12, 24 and 48 h, and kidney lesion parameters measured at 24 and 48 h in mice infected with 11 *C. albicans* strains.

	Cytokine/chemokine	Time (h)	Lesions/mm^2^				*C. albicans* pixels/lesion				Infiltrate pixels/mm^2^				log CFU/g kidney			
			24 h		48 h		24 h		48 h		24 h		48 h		24 h		48 h	
			*r* a	*F* b	*r*	*F*	*r*	*F*	*r*	*F*	*r*	*F*	*r*	*F*	*r*	*F*	*r*	*F*
Kidney	GM-CSF	12	−0.04	1.0	0.11	1.6	−0.06	0.1	0.01	0.3	0.02	0.5	−0.03	0.0	0.07	0.3	−0.18	0.0
		24	−0.39	6.7	−0.14	1.4	−0.15	0.8	−0.20	1.5	−0.26	2.6	−0.06	0.1	0.08	0.1	−0.25	2.2
		48	−0.29	3.6	−0.34	4.3	−0.36	4.4	−0.07	0.2	−0.06	0.1	−0.37	4.3	−0.37	5.1	−0.61*	18*
	IL-10	12	−0.23	0.9	−0.05	0.4	−0.16	0.8	−0.17	0.1	−0.25	0.1	0.08	1.3	0.14	0.1	−0.03	0.3
		24	−0.40	1.8	−0.20	0.0	−0.16	1.1	−0.22	0.1	−0.29	0.0	−0.09	0.3	0.08	0.4	−0.20	0.2
		48	−0.39	0.2	−0.38	2.6	−0.30	3.2	−0.31	1.5	−0.29	0.5	−0.25	0.9	−0.14	2.0	**−0.50**	**8.2**
	IL-6	12	0.38	1.0	0.31	0.8	0.03	6.8	0.15	0.6	−0.02	**8.1**	0.21	0.6	0.02	**9.6**	0.29	0.3
		24	0.85*	36*	0.55*	3.8	0.77*	44*	0.41	0.3	0.75*	27*	0.24	0.5	0.68*	73*	0.63*	7.4
		48	0.58*	1.8	**0.43**	1.1	**0.40**	0.6	0.77*	30*	0.68*	7.4	0.14	0.0	0.06	20*	**0.46**	0.8
	KC	12	0.69*	0.3	0.72*	6.9	**0.42**	1.2	0.32	0.0	0.37	1.1	0.55*	4.7	**0.49**	0.8	0.68*	2.0
		24	0.85*	15*	0.67*	0.1	0.66*	16*	**0.41**	0.0	0.61*	6.9	**0.48**	1.3	0.70*	33*	0.76*	2.0
		48	0.66*	0.9	0.62*	3.2	0.35	1.3	0.53*	4.5	**0.48**	0.3	0.62*	**9.2**	0.22	**12**	0.74*	**7.8**
	MIP-1b	12	−0.30	**11**	−0.30	**8.4**	−0.13	4.8	−0.26	0.0	−0.17	1.5	−0.21	3.3	0.02	**12**	**−0.41**	18*
		24	0.40	**11**	0.36	**8.3**	**0.49**	**11**	0.03	2.4	0.34	2.2	0.29	3.8	0.67*	43*	0.33	**12**
		48	0.68*	**13**	0.57*	5.2	**0.50**	2.6	0.77*	42*	0.71*	17*	0.40	1.3	0.18	2.5	0.61*	6.8
	MIP-2^c^	12	0.41	0.7	**0.56**	5.4	0.15	0.8	0.32	0.6	0.04	2.1	0.53	7.2	0.14	1.4	**0.59**	**11**
		24	0.89*	25*	**0.69**	2.4	0.77*	**10**	0.40	1.0	0.71*	**11**	0.38	0.9	0.81*	**15**	0.72*	2.0
		48	**0.64**	1.7	**0.64**	3.7	**0.67**	2.5	0.23	0.0	0.52	0.2	**0.61**	8.9	**0.67**	2.5	0.75*	**13**
	RANTES	12	0.31	2.5	**0.43**	5.8	0.31	2.8	0.35	4.2	0.32	3.0	0.21	0.7	0.18	0.5	0.30	1.9
		24	0.20	0.7	0.21	1.2	0.15	0.7	−0.03	0.5	0.10	0.1	0.31	3.0	**0.41**	7.2	0.36	3.7
		48	0.04	0.0	−0.14	1.5	−0.15	1.3	0.10	0.4	0.04	0.0	−0.08	0.8	−0.13	2.2	0.01	0.2
	TNF	12	−0.15	4.1	0.10	0.0	−0.17	**7.8**	−0.01	0.1	−0.24	7.3	0.12	0.0	0.09	1.6	0.07	0.0
		24	0.66*	18*	**0.48**	6.7	0.71*	31*	0.28	0.0	0.60*	**14**	0.27	3.7	0.78*	51*	**0.50**	5.4
		48	0.59*	5.1	0.19	0.0	**0.45**	0.5	0.59*	**11**	0.66*	**11**	−0.12	2.2	0.21	2.9	0.33	0.5
	G-CSF	12	0.33	0.6	0.38	4.4	0.17	0.2	0.11	0.0	0.20	0.0	0.12	1.1	0.13	0.5	0.17	0.0
		24	0.73*	**11**	0.54*	0.4	0.65*	**10**	0.53*	1.7	0.68*	**8.8**	0.18	1.9	0.52*	**10**	0.55*	2.3
		48	0.63*	5.9	0.68*	16*	**0.50**	1.4	0.70*	**13**	0.63*	6.0	0.57*	**15**	0.18	0.9	0.63*	**7.9**
	IL-1b	12	−0.15	1.4	−0.19	1.3	0.02	0.0	−0.04	0.0	0.08	0.3	−0.05	0.3	0.08	0.3	−0.16	1.9
		24	0.57*	**10**	0.36	2.5	0.57*	**13**	0.28	1.5	**0.49**	**8.6**	0.17	0.0	0.61*	**14**	0.41	1.8
		48	0.29	0.4	0.19	0.3	0.18	0.3	0.16	0.1	0.17	0.2	0.28	1.8	0.25	0.0	**0.42**	4.1
	MCP-1	12	0.20	0.1	**0.46**	4.2	0.26	0.0	0.16	0.8	0.16	0.0	0.30	1.1	0.30	0.5	0.41	2.7
		24	0.63*	5.0	0.54*	0.5	0.53*	6.9	0.14	0.6	**0.45**	3.6	**0.52**	0.1	0.74*	47*	0.62*	0.9
		48	0.60*	3.2	0.54*	4.3	0.28	0.3	0.31	3.0	0.31	0.0	0.66*	**10**	0.22	**10**	0.68*	**10**
	MIG	12	0.01	3.2	0.30	0.4	0.19	0.0	0.03	0.2	0.20	0.0	0.14	0.0	0.15	0.3	0.14	0.4
		24	0.55*	16*	**0.48**	5.3	0.54*	**11**	0.20	1.0	**0.46**	6.0	0.36	3.1	0.58*	21*	0.54*	**11**
		48	0.21	1.0	**0.14**	0.1	−0.03	0.5	0.28	2.0	0.18	0.4	0.20	0.7	−0.28	**7.8**	0.19	0.5
Spleen	GM-CSF	12	−0.54	**11**	−0.35	3.3	−0.27	1.6	−0.28	2.2	−0.25	1.2	−0.32	2.8	−0.22	1.0	**−0.45**	6.3
		24	−0.24	1.1	−0.13	0.0	−0.23	0.5	−0.11	0.1	−0.26	1.0	−0.01	0.3	−0.20	0.6	−0.09	0.0
		48	0.10	0.0	0.34	2.8	0.39	3.8	0.04	0.0	0.30	1.9	0.27	2.0	0.19	0.5	0.27	1.6
	IL-10	12	−0.15	0.7	−0.16	0.7	−0.06	0.1	−0.15	0.6	0.05	0.1	−0.21	1.2	0.03	0.0	−0.23	1.5
		24	0.19	1.0	−0.03	0.1	0.07	0.1	0.13	0.5	0.03	0.0	−0.17	1.3	−0.09	0.2	−0.15	1.0
		48	0.21	1.2	**0.44**	6.8	0.34	3.8	0.09	0.2	0.29	2.6	0.41	6.0	0.14	0.6	**0.41**	6.0
	KC	12	**0.49**	0.2	**0.50**	2.2	0.40	0.1	0.22	0.2	0.38	0.1	0.36	0.5	**0.41**	0.1	0.57*	3.0
		24	0.79*	26*	**0.53**	1.4	0.67*	14*	0.24	0.1	0.62*	**10**	0.41	0.2	0.76*	32*	0.66*	4.3
		48	**0.46**	1.4	**0.53**	3.0	0.28	0.0	0.27	0.7	0.31	0.1	0.56*	6.3	0.17	2.1	0.63*	6.1
	MIP-1b	12	0.23	1.3	−0.05	0.1	0.00	0.0	0.05	0.0	0.01	0.0	−0.18	0.9	−0.10	0.7	−0.06	0.3
		24	**−0.44**	7.5	−0.27	2.3	−0.33	3.6	−0.32	3.1	−0.33	3.6	−0.13	0.6	−0.13	0.6	−0.28	2.6
		48	−0.28	2.6	0.01	0.0	−0.11	0.4	−0.04	0.0	−0.14	0.6	0.13	0.3	−0.24	2.1	−0.13	0.6
	RANTES	12	0.00	0.0	0.18	0.7	0.18	1.0	0.11	0.4	0.11	0.5	0.14	0.4	0.12	0.2	0.26	1.1
		24	−0.10	0.9	0.08	0.0	−0.03	0.3	−0.10	0.6	−0.11	0.9	0.11	0.1	0.08	0.0	0.26	1.2
		48	−0.55*	14*	−0.22	1.1	−0.28	2.3	−0.21	1.3	−0.30	3.0	−0.13	0.4	−0.30	2.7	−0.30	2.2
	TNF	12	−0.29	3.0	−0.24	2.2	−0.26	2.3	−0.19	1.1	−0.14	0.7	−0.19	1.4	−0.05	0.1	−0.34	5.3
		24	−0.10	0.2	−0.25	1.8	−0.15	0.6	0.09	0.5	−0.20	0.9	−0.29	1.9	−0.26	1.4	−0.31	2.9
		48	−0.21	1.2	−0.18	0.5	−0.15	0.5	−0.23	2.1	−0.17	0.6	−0.29	2.0	−0.28	1.8	−0.32	2.9
	G-CSF	12	−0.26	2.0	−0.22	1.1	−0.21	1.3	−0.19	0.8	−0.13	0.3	−0.19	0.8	−0.04	0.1	−0.32	3.0
		24	0.37	3.3	0.21	0.5	**0.46**	6.5	0.26	0.9	**0.40**	4.3	−0.08	0.8	0.25	2.1	0.16	0.2
		48	**0.44**	5.4	**0.44**	5.4	**0.44**	5.2	0.61*	14*	0.59*	**13**	0.36	4.6	−0.02	0.2	**0.44**	5.8
	IL1-b	12	−0.02	0.7	0.02	0.2	0.09	0.0	−0.05	0.1	0.13	0.4	0.04	0.2	0.10	0.0	0.14	0.0
		24	0.68*	24*	**0.45**	4.6	**0.45**	6.3	0.34	5.6	**0.48**	**9.1**	0.27	0.5	0.32	2.7	**0.51**	5.0
		48	0.19	0.1	0.31	0.9	0.17	0.1	−0.08	1.6	0.04	0.6	0.39	3.5	0.14	0.0	**0.53**	5.4
	IL-9	12	−0.37	5.0	−0.21	1.2	**−0.46**	**9.5**	−0.26	1.8	**−0.40**	6.9	0.08	0.2	−0.25	2.4	−0.16	1.0
		24	0.14	0.6	−0.18	0.9	0.09	0.0	−0.28	1.6	0.10	0.0	−0.10	0.7	0.09	0.0	−0.01	0.4
		48	0.07	0.0	−0.04	0.1	0.21	2.1	−0.15	0.0	0.21	1.8	0.10	0.6	0.18	1.0	0.22	2.1
	MCP-1	12	**−0.50**	**11**	−0.20	1.1	−0.14	0.6	−0.29	2.5	−0.27	2.4	−0.19	0.9	0.01	0.0	−0.21	1.3
		24	0.30	3.2	0.20	0.9	0.38	4.3	−0.12	0.2	0.25	1.9	0.29	2.0	0.61*	15*	0.35	3.3
		48	**0.41**	4.4	0.26	1.1	0.24	0.8	−0.04	0.1	0.20	0.5	0.32	1.9	0.32	2.1	0.38	2.7
	MIG	12	−0.11	0.1	−0.23	0.7	−0.17	0.2	0.13	0.3	−0.22	0.1	−0.33	1.6	−0.29	0.0	−0.24	0.0
		24	0.40	5.2	**0.45**	6.4	0.54*	**12.3**	0.07	0.3	**0.43**	6.0	0.27	1.5	0.54*	**13**	**0.46**	7.4
		48	−0.21	1.1	−0.11	0.0	−0.31	3.0	0.10	0.0	−0.24	0.8	−0.24	0.2	**−0.42**	5.0	−0.38	3.4

a *r* = Pearson correlation coefficient.

b *F* = F-ratio from univariate analysis of variance.

c data for mice infected with 8 of the 11 *C. albicans* isolates.

All data presented to two significant figures. Boldface type indicates *p*<0.01; an asterisk indicates *p*<0.001.

From the overview of associations provided in Table 4 it can be seen that the earliest strong association between immune effector concentrations and kidney lesion parameters were for KC concentrations in the kidneys at 12 h, which significantly associated with 24 h data for lesion densities, *C. albicans* pixels, infiltrate pixels and viable burdens. Kidney levels of MIP-1β and IL-6 at 12 h also correlated, but less strongly than for 12 h KC levels, with two lesion parameters at 24 h.

**Table 4:** Association scores showing the strength of correlation and ANOVA data between concentrations of cytokines or chemokines and parameters of *C. albicans* kidney lesions.

	Cytokine/chemokine	Time (h)	Lesions/mm^2^		*C. albicans* pixels/lesion		Infiltrate pixels/mm^2^		log CFU/g kidney	
			Association score		Association score		Association score		Association score	
			24 h	48 h	24 h	48 h	24 h	48 h	24 h	48 h
Kidney	IL-6	12	0	0	0	0	1	0	1	0
		24	4	2	4	0	4	0	4	2
		48	2	1	1	4	2	0	2	1
	KC	12	2	2	1	0	0	2	1	2
		24	3	2	4	1	2	1	4	2
		48	2	2	0	2	1	3	1	3
	MIP-1β	12	1	1	0	0	0	0	1	3
		24	1	1	2	0	0	0	4	1
		48	3	2	1	4	4	0	0	2
	MIP-2	12	0	1	0	0	0	0	0	2
		24	4	1	3	0	3	0	3	2
		48	1	1	1	0	0	1	1	3
	TNF	12	0	0	1	0	0	0	0	0
		24	4	1	4	0	3	0	4	1
		48	2	0	1	3	3	0	0	0
	G-CSF	12	0	0	0	0	0	0	0	0
		24	3	2	3	2	3	0	3	2
		48	2	4	1	3	2	3	0	3
	IL-1β	12	0	0	0	0	0	0	0	0
		24	3	0	3	0	2	0	4	0
		48	0	0	0	0	0	0	0	1
	MCP-1	12	0	1	0	0	0	0	0	0
		24	2	2	2	0	1	1	4	2
		48	2	2	0	0	0	3	1	3
	MIG	12	0	0	0	0	0	0	0	0
		24	4	1	3	0	1	0	4	3
		48	0	0	0	0	0	0	1	0
Spleen	KC	12	1	1	0	0	0	0	1	2
		24	4	2	4	0	3	0	4	2
		48	1	1	0	0	0	2	0	2
	G-CSF	12	0	0	0	0	0	0	0	0
		24	0	0	1	0	1	0	0	0
		48	1	1	1	4	3	0	0	1
	IL-1β	12	0	0	0	0	0	0	0	0
		24	4	1	1	0	2	0	0	1
		48	0	0	0	0	0	0	0	1
	MIG	12	0	0	0	0	0	0	0	0
		24	0	1	3	0	1	0	3	1
		48	0	0	0	0	0	0	1	0

The data in Table 3 have been reduced by the following procedure. Cytokines or chemokines that showed statistically significant scores (p<0.01) with 3 or fewer lesion parameters across all 48 h of the experiment were removed from the tabulation. For the rest, a score of 1 was assigned for each *r* and *F* in Table 3 that was significant at the level of *p*<0.01, and a score of 2 for each *r* and *F* in Table 3 that was significant at the level of *p*<0.001. For each cross-tabulation of cytokine or chemokine with a lesion parameter the maximum possible score is therefore 4.

In general, 24 h cytokine concentrations in the kidney showed the highest level of statistical association with kidney lesions (Table 4), with 24 h measurements of IL-6, KC, MIP-2, TNF, G-CSF, IL-1 β, MCP-1 and MIG significantly correlated with all 4 lesion parameters at 24 h. Many of the correlations also remained significantly correlated with 48 h lesion parameters (Tables 3 and 4). G-CSF alone showed a higher level of significant correlation between its kidney concentration at 48 h and 48 h lesions. For the other chemokines and cytokines, kidney concentrations at 48 h showed less or no correlation with lesion parameters. These data were consistent with a temporal pattern of immune effector production in the kidneys in response to *C. albicans* challenge in which levels of most effectors correlated with number and severity of lesions, with KC, MIP-1 β and IL-6 the earliest lesion-correlated molecules to appear and G-CSF the latest.

For GM-CSF and IL-10 the sign of the correlation coefficients was predominantly negative (Table 3), indicating that higher levels of these two effectors were associated with lower levels of kidney damage. For all other chemokines and cytokines measured in the kidneys the correlations were positive

An unexpected finding was a correlation between levels of some immune effectors in the spleen and lesions in the kidney. Spleen KC, at all three sample times, showed statistically significant correlations with at least two of the kidney lesion parameters at 24 and 48 h (Tables 3, 4), with a particularly strong association between 24 h spleen KC concentrations and 24 h kidney lesions. There was also significant association between 24 h spleen concentrations of both IL-1 β and MIG and three of the four kidney lesion measurements (Table 4), while 48 h spleen levels of G-CSF correlated with 48 h lesion measurements.

**Figure 8:**
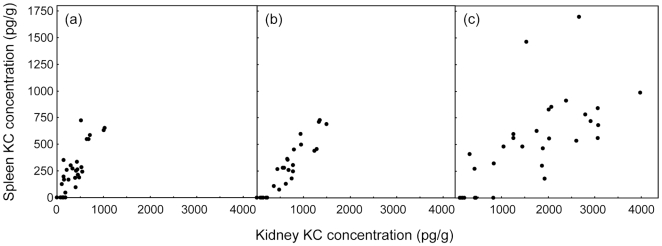
Scatterplots of KC concentrations in kidney vs. spleen measured at (a) 12 h, (b) 24 h and (c) 48 h after intravenous challenge of mice with 11 *C. albicans* strains.

No other spleen measurements of cytokine or chemokine concentrations were so consistently associated with kidney lesion parameters. The clear association of spleen KC levels with all four lesion parameters prompted us to analyze the associations between spleen and kidney KC levels. The results (Table 5) unequivocally demonstrate the strongest correlations were between KC concentrations measured in the two tissues at the same time. Fig. 8 shows the raw data for these strongest correlations as scatterplots. At each measurement time, KC levels in kidneys were approximately twice as high as those in spleen samples.

**Table 5:** Associations between KC concentrations in kidney and spleen.

Kidney KC at	vs Spleen KC at 12 h		vs Spleen KC at 24 h		vs Spleen KC at 48 h	
	*r*	*F*	*r*	*F*	*r*	*F*
12 h	0.85*	42*	0.628*	6.8	0.313	0.51
24 h	0.64*	4.9	0.939*	140*	0.596*	**8.9**
48 h	0.40	0.54	**0.445**	0.94	0.701*	19*

Data are presented in the same format as Table 3.

## Discussion

This study demonstrates that, in the mouse intravenous *C. albicans* challenge model, early interactions between the fungus and host predict the level of gross progression of disease in the kidney, the major organ affected in the model [9], [11]. Our study is the first we are aware of to link cytokine or chemokine production with refined histopathological measurements of lesion development in the mouse model. Because we measured immune effector concentrations at different stages in the progression of renal disease, we can hypothesize the sequence of activation of these molecules from the statistical associations between their concentrations and the four parameters relating to lesion development in the kidney.

The significant and ubiquitous association of 12 h kidney KC concentrations with subsequent kidney lesion parameters (Tables 3, 4) strongly implicates this chemokine, the murine analog of human CXCL8/IL-8, which works in conjunction with MIP-2, as an important, early produced factor in the development of the host response to *C. albicans* in kidney parenchyma. Its main role is as a chemoattractant in mobilization of leukocyte infiltrates. A recent mouse study showed that resident tissue macrophages are the main source of KC and MIP-2 [17], although all types of kidney cells are able to express cytokines and chemokines *in vitro* [18]. KC and MIP-2 are both transcriptionally regulated by signalling through TLR2, TLR3 and TLR4 [17]; with TLR2 and TLR4 well established as primary receptors in recognition of *C. albicans* polysaccharides and susceptibility to experimental systemic *C. albicans* infection [16], [19]. The effect of KC and MIP-2 production is “robust neutrophil recruitment” to the site of infection [17]. We therefore hypothesize that KC production, a result of the interaction between *C. albicans* surface mannans and glucans and toll-like receptors, is a critical early event that mobilizes the host infiltrates seen in the kidney.

Among the many host-fungus correlations of interest in this study is the consistent finding that more severe disease (higher lesion densities, *C. albicans* pixels and viable tissue burdens), resulting from the most virulent challenge strains, associates with higher levels of host leukocyte infiltrates (Table 2), as well as with kidney levels of many cytokines and chemokines (IL-6, KC, MIP-1β, MIP-2, TNF, G-CSF, MCP-1 and MIG dominate the list in Table 4). We consider these results suggest that a heightened host (innate) immune response to *C. albicans* in the mouse model may worsen kidney pathology by generating inflammation, rather than protecting the host. A very extensive literature, dating back many years, defines innate immune responses based on polymorphonuclear leukocytes (PMNs) and macrophages as the principal cellular elements of defense against *C. albicans* invasion, responsible for phagocytosis and intracellular killing of the fungi (examples of reviews are [16], [19], [20]). However, as was first shown [21] and confirmed [22] many years ago, virulent *C. albicans* isolates are more likely than others to kill and escape from PMNs *in vitro*. The greater influx of PMNs we measured in the kidneys of mice infected with more virulent isolates may be associated with difficulties of eradicating the fungus in the kidney, with products from destruction of PMNs contributing to local tissue damage.

It has been previously acknowledged that the balance in innate responses between (beneficial) fungicidal immunity and (detrimental) inflammatory cytotoxicity determines levels of tissue damage in fungal infections [19]. Moreover, the generally adverse pathological role of inflammatory responses to many types of infection has been recognized since the 1970s [13]. However, the emphasis of research into anti-*Candida* phagocytic function by host leukocytes has assumed this function is purely defensive. While innate responses have been shown to contribute to pathological changes in the human vagina in experimental *C. albicans* challenge experiments [23], relatively few studies have generated direct evidence for any tissue-damaging role of host innate immune responses in the mouse disseminated *C. albicans* infection model [24]. The view of phagocytosis purely as a host defense against *C. albicans* infection is challenged by the empirical observation of micro-abscess formation in kidneys [12]: our data show the host-fungus interaction in the *C. albicans* mouse challenge model is comparable with that well-known for bacterial infections in which phagocytic processes contribute to host damage [14].

The evidence from the present study shows unequivocally that kidney tissue damage is related *quantitatively* to the level of the host innate response. This finding is also in accordance with the growing body of evidence showing lowered cytokine production from monocytes and PMNs stimulated *in vitro* with virulence-attenuated *C. albicans* mutants bearing deficient or abnormal cell-wall polysaccharides [16], [25]–[27]. If innate responses have a purely defensive role, then such reduced cytokine production should result in greater fungal proliferation *in vivo*. From the evidence of the present study, *C. albicans* strains naturally attenuated in gross virulence for mice, including the mannan-depleted mutant *pmr1Δ*, do not induce a strong innate immune response at the early stages of infection. The studies just cited are examples of those exploring the detailed interaction of *C. albicans* surface macromolecules with host cell receptors *in vitro*, with toll-like receptors and dectin-1 shown to be those most involved in the fungal pathogen recognition process.

Our findings concerning the chemokines and cytokines produced during experimental *C. albicans* infections agree well with those of other studies. Yamamoto *et al*. [28] showed that levels of mRNAs encoding IL-1β, IL-6, GM-CSF, MIP-1β, MIP-2, and KC rose in macrophages exposed to *C. albicans in vitro*. They also showed these changes were regulated via the macrophage mannose receptor [28], now known as the site for specific binding of *N*-linked *C. albicans* cell wall mannans [27]. We also found increases in the kidney levels of IL-1β, IL-6, MIP-1β and KC in our model (Figs. 4–6).

The tissue specificity of mouse cytokine responses to disseminated *C. albicans* infection has been demonstrated before [10]. However, KC was not only the earliest cytokine in the kidney whose levels gave the best statistical correlation with all four lesion parameters, it was also produced by 12 h in the spleen. By 24 h, spleen KC concentrations correlated very strongly with 24 h estimates of lesion density, *C. albicans* pixels, infiltrate pixels and viable burdens *in the kidney* (Tables 3, 4). These associations led us to correlate levels of KC in spleen and kidney samples directly (Table 5, Fig. 8). There were very strong associations between KC levels in kidney and spleen at each sample time, with kidney levels approximately twice those in spleen samples. We interpret these results as indicating either that KC produced in the kidney enters the circulation and is therefore detectable in the spleen but at lower levels, or that KC is produced in both organs in proportionate response to *C. albicans* infection, with the spleen generating less KC than the kidney. Our experimental design did not include measurement of serum cytokine and chemokine levels, since our interest was in local responses to infection in one organ that ultimately clears infection and another in which infection progresses.

Apart from KC, IL-6 and MIP-1β were the only other cytokine and chemokine with 12 h kidney levels that correlated with two or more lesion parameters at 24 h (Tables 3, 4). Expression of IL-6 has been previously associated with recruitment of neutrophils to the site of *C. albicans* infection in mice, and animals genetically deficient in IL-6 showed enhanced susceptibility to IV *C. albicans* challenge [29], [30]. Comparison of mice lacking genes encoding both G-CSF and IL-6 with their counterparts lacking G-CSF showed greater susceptibiliy to *C. albicans* challenge in the former [31]. While these data all suggest a protective role for IL-6 in experimental murine *C. albicans* infections, it is notable that IL6 ^—/—^ mice rendered neutropenic before *C. albicans* challenge had 72 h viable organ burdens indistinguishable from those of neutropenic control animals and higher than in mice with normal neutrophil counts [29]. Hence, in all situations the development of tissue pathology in *C. albicans*-infected mice appears to depend very much on relative balances of cytokine or chemokine production and of neutrophil recruitment to the kidney. Some degree of response involving cytokine expression and neutrophil recruitment is clearly required for host defense against *C. albicans*: perhaps a modest acute host response is more successful in eradicating the fungus and avoiding progressive kidney damage than a more florid host reaction in the kidney.

Progressive disease in the mouse *C. albicans* model has long been associated with a predominantly Th2-type, rather than a Th-1-type host response. More recently, the later involvement of a Th17 response in murine *C. albicans* infection has been implicated (reviewed in [8]). However, the contribution of a Th17 response to exacerbation of *C. albicans* burdens in a gastric model of *Candida* infection [32] has to be contrasted with the unequivocally protective effect of the Th17 response in disseminated murine *C. albicans* infection [33]. Our own data showed little or no renal production of cytokines or chemokines classically associated with a Th1 response in association with *C. albicans* kidney proliferation. IL-12, IFNγ and, for that matter, IL-2, IL-3, IL-4, IL-5, IL-9, IL-13 and even IL-17 were essentially undetectable in the great majority of our samples. By contrast, G-CSF, TNF, and MCP-1 levels in the kidneys at 24 and 48 h showed associations with lesion parameters at both times (Tables 3, 4), possibly indicating involvement of these effectors in the progression of infection subsequent to the neutrophil-recruiting early activities of KC, IL-6 and MIP-1β. Significant associations between kidney cytokine or chemokine levels and measurements of both infiltrate and *C. albicans* pixels by histopathology were seen 24 h after challenge (Table 4). By contrast, associations between immune effector levels and overall lesion densities and viable cell numbers were more commonly retained at 48 h. This observation is consistent with our interpretation of the overall outcome of infection depending on the earliest stages of host-fungus interactions. By 48 h, neither the *C. albicans* pixels nor the amount of host infiltrate was as strongly correlated with lesion density or viable burden as at 24 h (Table 2), suggesting that the different 48 h parameters may reflect the outcomes of intra-lesional phagocytic processes.

Our results provide clear evidence from immunologically intact animals for the significance of the early host response in determining the pathological course of infection, and strongly implicate KC, in particular, as an important early mediator of overall outcome. However, we realize these findings are strictly applicable only to infections in immunologically intact BALB/c mice. Since other standard inbred mouse strains differ in their susceptibility to *C. albicans* challenge, a phenomenon related to the mouse genes Carg1 and Carg2 [34], [35], our study serves as a basis for future expansion of research into different mouse strains, including mice with gene knockouts specific for the cytokines we have identified as significant early factors in determining infection outcome, and mice immunocompromised by other experimental approaches. Correlation of individual cytokine responses in different tissues with the fine detail of lesion development as we have done in this study will, in time, provide evidence for the precise nature of the main immunostimulatory fungal components and of the innate immune responses to them within the precise context of whole-animal infection. Such studies will usefully complement reductionist investigations of single component interactions *in vitro*.

## Methods

### Ethics statement

All animal experimentation conformed with the requirements of UK Home Office legislation for animal experimentation, and with those of the University of Aberdeen Ethical Review Committee.

### *C. albicans* strains

Eleven strains of *C. albicans* were used in the experiments. They are detailed in Table 1. Nine of the strains were chosen to represent the four major clades of *C. albicans* strain types [36], with one high virulence and one and low virulence strain chosen from each clade from among 43 isolates tested in a separate study [37]. The other two strains were NGY355 and NGY356; the former a mutant with disruptions in both copies of *PMR1*, the latter a heterozygous *PMR1* reintegrant [38]. These two strains will be referred to as *pmr1*Δ and *pmr1/PMR1* throughout this paper. They were included as representatives of laboratory-engineered mutants of different virulence to determine if their behavior *in vivo* differed notably from wild-type strains.

All strains were maintained in 50% glycerol stocks at −80°C and recovered from these stocks as required for experiments. For routine maintenance in culture, the fungi were grown on slants of Sabouraud agar (Oxoid, Basingstoke, UK). For preparation of inocula for mouse challenge, a yeast culture was grown for 18 h in 5 ml volumes of NGY medium, comprising 0.1% Neopeptone (BD-Difco, Oxford, UK), 0.4% glucose and 0.1% yeast extract (BD-Difco), incubated at 30°C with continuous rotation at 20 rpm and an angle of 5° from the horizontal. These conditions yielded a suspension of yeast cells in late exponential growth phase. The yeasts were centrifuged and washed twice in sterile saline, resuspended in saline, and enumerated with a hemocytometer. Pairs of cells, as well as rare triplets and quadruplets, were counted as single units, to maximize compatibility between hemocytometer counts and viable counts, set up from the final inoculum suspensions and enumerated the following day.

### Animal model

Female BALB/c mice (Harlan, UK), weighing 18–22 g, were supplied with food and water *ad libitum*. They were weighed and infected intravenously with the *C. albicans* isolates at a target dose of 3×10^4^ CFU/g body weight. Viable counts showed the animals received doses ranging from 1.8×10^4^–5.6×10^4^ CFU/g, with the median and modal doses both 3.6×10^4^ CFU/g. Groups of nine animals were challenged for each strain, then 3 mice were humanely terminated after 12 h, 24 h and 48 h. A pilot experiment was done with strains SC5314, HUN96, J990102 and AM2003/0074 to allow us to develop and validate the methodology used for lesion measurements and cytokine assays. The experiment was then repeated with all 11 *C. albicans* strains, and results from this experiment are presented in this paper. Data for each time point were therefore obtained from 3 animals for each of 11 infecting strains, with the exception of some cytokine measurements (see below). A group of control mice was injected IV with 100 µl saline in each experiment.

At termination, each animal was swiftly dissected under aseptic precautions. Both kidneys and the spleen were removed. The organs were halved longitudinally with a sterile scalpel. Two halves from each kidney were pooled and homogenized in 0.5 ml of sterile saline containing one Complete Protease Inhibitor Tablet (Roche) per 50 ml. The other two kidney halves were fixed together in 4% formaldehyde to be processed for histopathology. The spleen halves were handled similarly: one half was homogenized in 0.5 ml saline containing protease inhibitor and the other fixed in formaldehyde. Weights of tissues were determined for the homogenized samples to provide a denominator for viable count and cytokine level data.

Samples (50 µl) of kidney and spleen homogenates were used for determination of tissue *C. albicans* burdens by viable counting. (No *C. albicans* was recovered from any sample from mice challenged with saline alone.) The remainder of the homogenate was centrifuged at 2500 g for 5 min, and the supernatant stored at −20°C for subsequent assay of cytokine concentrations.

### Cytokine assays

Twenty mouse cytokines and chemokines were assayed in supernatants from tissue homogenates by means of BD cytometric bead array (CBA) (BD, Oxford, UK). The cytokines measured were IFN-γ, G-CSF, GM-CSF, IL-1β, IL-2, IL-3, IL-4, IL-5, IL-6, IL-9, IL-10, IL-12p70, IL-13, IL-17, and TNF. The chemokines measured were KC, MCP-1, MIG, MIP-1β, and RANTES. Additionally, MIP-2 was determined by immunoassay in kidney homogenates from animals infected with 8 of the 11 *C. albicans* isolates (see below).

For each neat sample two CBA flex set reactions were set up, each measuring ten different cytokines/chemokines. Reactions were carried out as per the manufacturer's instructions. CBA reactions were analysed by flow cytometry on a FACSArray (BD, UK). To analyze results, discrete bead populations were selected and labelled with FCS Filter software v1.0.1 (www.softflow.com) and concentrations of cytokines and chemokines determined from standard curves with FCAP Array software v1.0.1 (www.softflow.com). Standard curves (range: 10–2500 pg/ml) were determined in the same run as the assayed samples. Homogenate samples close to, or exceeding, the maximum point of the standard curve were diluted and assayed again. For measurement of MIG, samples were routinely diluted 1 in 5.

Murine IFN-γ, IL-17 and IL-12p70 were assayed with Quantikine® mouse immunoassays (R & D Systems, Abingdon, UK) to confirm the negative results obtained in bead assays, and MIP-2 was assayed for kidney samples from mice infected with 8 of the 11 isolates, to extend the range of chemokines tested. Assays were conducted according to the manufacturer's instructions, with undiluted supernatants (50 µl) from kidney and spleen homogenates obtained from *C. albicans*-infected BALB/c mice. Kidney homogenates from two uninfected mice were also spiked with either IFN-γ or IL-12p70 (80–100 pg/ml) and the supernatants assayed for cytokines.

The concentration of each cytokine was determined in units of pg/ml, then recalculated as pg/g tissue in the sample. The effective limit of detection for the cytokines was 25 pg/ml: samples determined with concentrations below this level were recorded as containing 0 pg/ml. The mean pg/g for each cytokine determined in the equivalent tissues from mice challenged with sterile saline was subtracted from the values for tissues from *C. albicans*-infected mice to provide a final cytokine concentration resulting from infection. A negative value was interpreted as probably indicating downregulation of the cytokine measured. Cytokine and chemokine measurements at each time point were therefore based on N = 33 (11 strains, triplicate mice).

Supplementary on-line table S1 provides the detailed values for all lesion and cytokine measurements.

### Semiquantitative histopathology

Two kidney halves from each animal were fixed in formaldehyde then embedded together in paraffin and sectioned longitudinally at 5 µm thickness. For each animal/timepoint, kidney lesions were subjected to semi-quantitation as described below for one section made from the first 20 µm depth of the embedded kidney and a second section made 80 µm below the first section.

Sections were deparaffinized, then stained with periodic acid-Schiff's reagent (PAS) and counterstained with hematoxylin. This method provided sections in which *C. albicans* elements within lesions were visible as red-stained, PAS-positive material, while host leukocyte infiltrates were visualized as a darker, purple color (Fig. 1). Adobe Photoshop CS3 software was used to determine pixel areas of lesions and of differently colored portions of lesions by means of the ‘magic wand tool’ set to a tolerance or 15–25 and for selection of non-contiguous pixels of matching color. Total pixel counts of selected areas were read from the Photoshop histogram plots at cache level 2. The tissue sections were photographed at a 1.2× magnification in a light box fitted with a digital camera for determination of entire sectional areas in mm^2^ from a predetermined pixel count calibration. The sections were then scrutinized microscopically with a 10x objective and individual lesions photographed with a digital camera. The lesional area in each photograph was outlined with the Photoshop ‘lasso tool’, cut and pasted into a new Photoshop window. The magic wand tool was then used to determine separately the pixel areas of PAS-positive material and of host infiltrate within each lesion. The spleen sections contained considerable amounts of non-fungal PAS-positive material, and defined lesions were difficult to discern. Similarly, in 12 h kidney specimens, well-defined lesions and *C. albicans* elements were rarely seen and seldom capable of pixel analysis. Our experience suggests that *C. albicans* elements are reproducibly visible in histopathological sections only when the viable counts in the tissues exceed 10^5^/g. In practice, we were able to obtain semi-quantitative lesion data only for sections of kidney taken from infected animals 24 h and 48 h after IV fungal challenge.

From the pixel data for each animal's kidneys after 24 and 48 h, the following determinations were made: lesion density (lesions/mm^2^  =  lesion count ÷ kidney area), *C. albicans* pixels/lesion (PAS-positive pixels in each *C. albicans*-positive lesion), and host infiltrate pixels/mm^2^. Comparison of these data for the two separate sections analyzed for each tissue block showed a high level of agreement between the two estimates. For each animal, the data from both sections were therefore pooled for determination of average estimates of lesion densities and pixel counts per kidney.

In the pilot experiment, lesion areas (in pixels) and infiltrate pixels per lesion were also determined, but these measurements were subsequently abandoned. It was found that determination of lesion areas involved too much subjective judgement of lesion boundaries to provide reproducible results when different operators assessed the same lesion photographs. While the color of PAS-positive material was sufficiently distinctive to allow reproducible assay of its pixel area per lesion, regardless of inter-operator variations in lesional area selected, the same was not true for the infiltrate pixel area. Expression of the total infiltrate pixels per mm^2^ of entire sections was found to provide better inter-operator reproducibility than was obtained for measurements of infiltrate pixels per lesion. Hence the parameters finally determined semi-quantitatively for analysis were lesions/mm^2^, infiltrate pixels/mm^2^, and PAS-positive pixels per *C. albicans*-positive lesion. Additionally, tissue burdens (as log_10_ CFU/g) were determined at the analysis time points.

### Statistical analysis

Data were stored in Microsoft Excel spreadsheets; the SPSS statistical package, version 17.0, was used for all analyses. The results available for analysis were concentrations of cytokines determined for triplicate animals at 12, 24 and 48 h in samples from kidneys and spleens infected with 11 strains of *C. albicans* (9 strains in some instances: see above), and the kidney lesion parameters — viable fungal kidney burdens at 12 h, 24 h and 48 h and estimates of lesion density, host infiltrate pixels/mm2 and PAS-positive (*C. albicans*) pixels per *Candida*-positive lesion, all at 24 h and 48 h. Comparisons of data were made on an individual animal basis.

Pearson's correlation coefficient, *r*, was used to determine associations between lesion data. For comparisons between lesion parameters and cytokine concentrations, many of the variables analyzed were unlikely to be fully independent of each other, so the following approach to statistical analysis was used. To determine associations between levels of each cytokine at each of 3 time points and lesion measurements at 2 time points, the 6 Pearson correlation coefficients for all possible lesion-cytokine pairs were calculated. Additionally, a univariate analysis of variance (ANOVA) was run with a lesion parameter at a single time point as dependent variable, and concentrations of cytokines at all three time points as covariates. From all analyses, a *p*-value ≤0.01 was regarded as a significant result and a *p*-value ≤0.001 as a highly significant result.

## Supporting Information

Table S1 Lists raw data on which analyses are based
(0.08 MB XLS)
